# Cryo-EM Structures of a Gonococcal Multidrug Efflux Pump Illuminate a Mechanism of Drug Recognition and Resistance

**DOI:** 10.1128/mBio.00996-20

**Published:** 2020-05-26

**Authors:** Meinan Lyu, Mitchell A. Moseng, Jennifer L. Reimche, Concerta L. Holley, Vijaya Dhulipala, Chih-Chia Su, William M. Shafer, Edward W. Yu

**Affiliations:** a Department of Pharmacology, Case Western Reserve University School of Medicine, Cleveland, Ohio, USA; b Department of Microbiology and Immunology, Emory University School of Medicine, Atlanta, Georgia, USA; c Emory Antibiotic Resistance Center, Emory University School of Medicine, Atlanta, Georgia, USA; d Laboratories of Microbial Pathogenesis, VA Medical Center, Decatur, Georgia, USA; Harvard Medical School

**Keywords:** cryo-EM, *Neisseria gonorrhoeae*, efflux pumps, multidrug resistance, structural biology

## Abstract

Neisseria gonorrhoeae is an obligate human pathogen and causative agent of the sexually transmitted infection (STI) gonorrhea. The most predominant and clinically important multidrug efflux system in N. gonorrhoeae is the multiple transferrable resistance (Mtr) pump, which mediates resistance to a number of different classes of structurally diverse antimicrobial agents, including clinically used antibiotics (e.g., β-lactams and macrolides), dyes, detergents and host-derived antimicrobials (e.g., cationic antimicrobial peptides and bile salts). Recently, it has been found that gonococci bearing mosaic-like sequences within the *mtrD* gene can result in amino acid changes that increase the MtrD multidrug efflux pump activity, probably by influencing antimicrobial recognition and/or extrusion to elevate the level of antibiotic resistance. Here, we report drug-bound solution structures of the MtrD multidrug efflux pump carrying a mosaic-like sequence using single-particle cryo-electron microscopy, with the antibiotics bound deeply inside the periplasmic domain of the pump. Through this structural approach coupled with genetic studies, we identify critical amino acids that are important for drug resistance and propose a mechanism for proton translocation.

## Abstract (precis)

Neisseria gonorrhoeae has become a highly antimicrobial-resistant Gram-negative pathogen. Multidrug efflux is a major mechanism that N. gonorrhoeae uses to counteract the action of multiple classes of antibiotics. It appears that gonococci bearing mosaic-like sequences within the gene *mtrD*, encoding the most predominant and clinically important transporter of any gonococcal multidrug efflux pump, significantly elevate drug resistance and enhance transport function. Here, we report cryo-electron microscopy (EM) structures of N. gonorrhoeae MtrD carrying a mosaic-like sequence that allow us to understand the mechanism of drug recognition. Our work will ultimately inform structure-guided drug design for inhibiting these critical multidrug efflux pumps.

## INTRODUCTION

Neisseria gonorrhoeae is a Gram-negative diplococcus, which infects humans and causes the sexually transmitted infection (STI) gonorrhea. Gonorrhea is one of the oldest described diseases and remains a significant global problem, with ca. 87 million cases reported annually worldwide (1). Antimicrobial resistance (AMR) is a major concern for the effective treatment of gonorrhea and threatens future clinical treatment regimens, especially if new antibiotics are not brought into clinical practice (2, 3). In 2018, the emergence of a “super drug-resistant” strain of N. gonorrhoeae was identified in the United Kingdom (4). Infection caused by this strain was refractory to treatment by azithromycin (Azi) and ceftriaxone (Cro), the two antibiotics recommended as the first-choices for dual treatment of gonorrhea.

Since N. gonorrhoeae is strictly a human pathogen and can colonize both male and female genital mucosal surfaces and other sites, it has developed various mechanisms to overcome the antimicrobial systems of the host innate immunity (5). The gonococcus employs a number of strategies to evade host attack. It possesses an intricate mechanism of antigenic variability through the differential expression of the genome (6). It is able to acquire new genetic material, cause asymptomatic infections, and readily develop resistance to antimicrobials (2, 3, 7). Invasive gonorrhea infections, which are often found in women, can provoke severe reproductive or general health complications. Furthermore, many gonorrheal infections, especially in women, are asymptomatic and can be silently spread to sexual partners and create serious future medical problems for these patients.

Multidrug efflux is considered one of the major causes of the failure of drug-based treatments of infectious diseases, which appears to be increasing in prevalence (8). In N. gonorrhoeae, the best characterized and most clinically important multidrug efflux system that mediates multidrug resistance (MDR) is the multiple transferrable resistance (Mtr)CDE tripartite efflux pump (9–16). This system recognizes and confers resistance to a variety of antimicrobial agents, including macrolides, β-lactams, cationic antimicrobial peptides, bile salts, dyes, and detergents (17). The *mtrCDE* locus consists of three tandemly linked genes encoding MtrC, MtrD, and MtrE, where all three components are absolutely required for substrate extrusion. This tripartite system comprises the MtrD inner membrane multidrug efflux pump and belongs to the resistance-nodulation-cell division (RND) superfamily of transport proteins (18), which constitutes substrate-binding sites and a proton-relay network to generate the proton-motive-force (PMF). MtrD works in conjunction with the MtrC periplasmic membrane fusion protein and MtrE outer membrane channel to actively export antimicrobials out of bacterial cells (9–16). Importantly, it has been shown that overexpression of the MtrCDE multidrug efflux pump contributes significantly to clinically relevant levels of resistance to β-lactams and macrolides (17).

An increasing amount of evidence suggests that a transfer of DNA from commensal *Neisseria* spp. into the *mtr* locus is capable of resulting in multiple nucleotide changes, which elevate gonococcal resistance to clinically important antibiotics, such as Azi and Cro (17, 19). Recently, Wadsworth et al. found that gonococci bearing mosaic-like sequences within *mtrD*, encoding the MtrD multidrug efflux pump, show strong linkage disequilibrium and epistatic effects that likely enhance the activity of the pump (20). To elucidate how MtrD carrying these mosaic-like sequences elevates drug resistance and enhances transport function, we decided to determine a cryo-electron microscopy (EM) structure of these efflux pumps. We chose to focus on full-length MtrD from the gonococcal strain CR.103, designated MtrD_CR103_, as this multidrug efflux pump has been shown to decrease the level of susceptibility for several antimicrobials, including erythromycin (Ery) and Azi (17). Although MtrD is similar to other members of RND transporters, such as AcrB (21, 22), AdeB (23), CmeB (24), and MexB (25), that also recognize and export multiple antimicrobials, differences in amino acid sequences exist and could influence structure-function relationships. Thus, while published structures for similar AcrB types of efflux transporters guided our work on MtrD (reviewed in reference 26), it was necessary to perform detailed structural and functional studies on MtrD to define regions of MtrD that contribute to antimicrobial resistance. Here, we present two solution cryo-EM structures of the N. gonorrhoeae MtrD_CR103_ multidrug efflux pump bound with hydrolyzed, decarboxylated ampicillin (Amp) and Ery at resolutions of 3.02 Å and 2.72 Å, respectively. Based on this structural information, we identified important drug-binding residues and modes of MtrD_CR103_-drug interactions. Both the Amp and Ery molecules bind at the distal drug-binding site in the periplasmic domain of MtrD_CR103_. The two substrate binding sites partially overlap each other, and the MtrD_CR103_ efflux pump utilizes slightly different subsets of amino acids to bind these two drugs. Our structural and functional studies indicate that the conserved charged residues R714 and E823 of MtrD_CR103_ are crucial for the recognition of macrolides and could provide clinical nonsusceptibility to Azi.

## 

### Structure of MtrD_CR103_.

We recently described the construction of genetic derivatives of antibiotic-sensitive N. gonorrhoeae strain FA19, which displays low level expression of a wild-type (WT) MtrCDE efflux pump, that contained amino acid replacements at the C-terminal end of MtrD (17). These amino acid changes were derived by the transformation of strain FA19 using donor DNA from an *mtrD*-mosaic clinical strain (CDC2), resulting in the replacement of the chromosomal copy of the WT gene. The MtrD protein of one of these transformants (CR.103) differed from that of FA19 by 23 amino acids and resulted in increased N. gonorrhoeae resistance to antimicrobials exported by MtrCDE; the amino acid differences between the WT MtrD possessed by strain FA19 and the MtrD variant expressed by CR.103 has been presented previously (17). Using a PCR-derived product from the 3′ end of the CR.103 *mtrD* sequence, we were able to obtain a transformant (CR.104) that had only two amino acid changes (S821A and K823E) compared with the wild-type FA19 sequence (19). To extend this work, we sought to determine if residues 821 or 823 or both were responsible for the antimicrobial resistance phenotype of transformant strain CR.104. Of these two amino acid changes, only the K823E change could increase the Azi resistance property (compared with that endowed by a WT *mtrD*) when present in a genetic derivative of strain FA19 lacking a functional MtrD transporter (Table 1). As an additional control, we constructed an S825A mutation, introduced it into strain KH14, and found that it, like the S821A change, did not increase antimicrobial resistance above WT levels (Table 1). Interestingly, S825 of MtrD corresponds in position to L828 in AcrB that is known to be important in forming the entrance binding site of AcrB. Thus, along with position 823, amino acid sequence differences between AcrB and MtrD may exert different influences on antimicrobial recognition and efflux.

**TABLE 1:** Antimicrobial susceptibility of MtrD site-directed mutants^*a*^

Strain by category	MIC^*b*^ (μg/ml) by treatment with:							
	Amp	Pen	Azi	Ery	EtBr	CV	Pmb	TX-100
KH14 background								
KH14	0.06	0.0075	0.06	0.06	0.5	0.03	50	25
WT	0.06	0.0075	0.25	0.5	2	1.25	200	100
KH14 R174Q	0.06	0.0075	0.25	0.5	**4**	1.25	200	100
KH14 E669G	0.06	0.0075	**0.125**	**0.25**	**1**	1.25	**100**	100
KH14 R714G	0.06	0.0075	**0.5**	**1**	2	1.25	200	100
KH14 S821A	0.06	0.0075	0.25	0.5	2	1.25	200	100
KH14 K823E	0.06	0.0075	**0.5**	**1**	2	1.25	200	100
KH14 S825A	0.06	0.0075	0.25	0.5	2	1.25	200	100
KH15 background								
KH15		0.25	1	2	8	5	400	
KH15 R714G		0.25	**8**	**8**	**16**	5	**1,600**	
KH15 K823E		0.25	**4**	**4**	8	5	**1,600**	

a Amp, ampicillin; Pen, penicillin; Azi; azithromycin; Ery, erythromycin; EtBr, ethidium bromide; CV, crystal violet; Pmb, polymyxin B; TX-100, Triton X-100.

b Antimicrobial susceptibility was determined by agar dilution. All assays are representative values from 3–9 assays. Bolded MIC values represent those at least 2-fold greater or less than that of the strain with a WT *mtrD* gene.

In order to determine the influence of overexpression of the *mtrCDE* efflux pump along with a single MtrD mutation that endowed increased antimicrobial resistance expressed by gonococci, the K823E mutation was introduced into a genetic derivative of FA19 that overexpresses the *mtrCDE* operon. For this purpose, we used a derivative of strain FA19 that has a single-base pair deletion in the *mtrR* promoter known to elevate *mtrCDE* expression and antimicrobial resistance (strain KH15 [9]). Importantly, the presence of the K823E mutation increased Azi resistance of KH15 by 4-fold (Table 1). Thus, N. gonorrhoeae carrying the K823E mutation would be classified as clinically nonsusceptible to Azi, as an official breakpoint for Azi is still under debate (27). Hence, the amino acid replacement at position 823 of MtrD is critical for the increased Azi-resistance property of mosaic strain CDC2.

**FIG 1:**
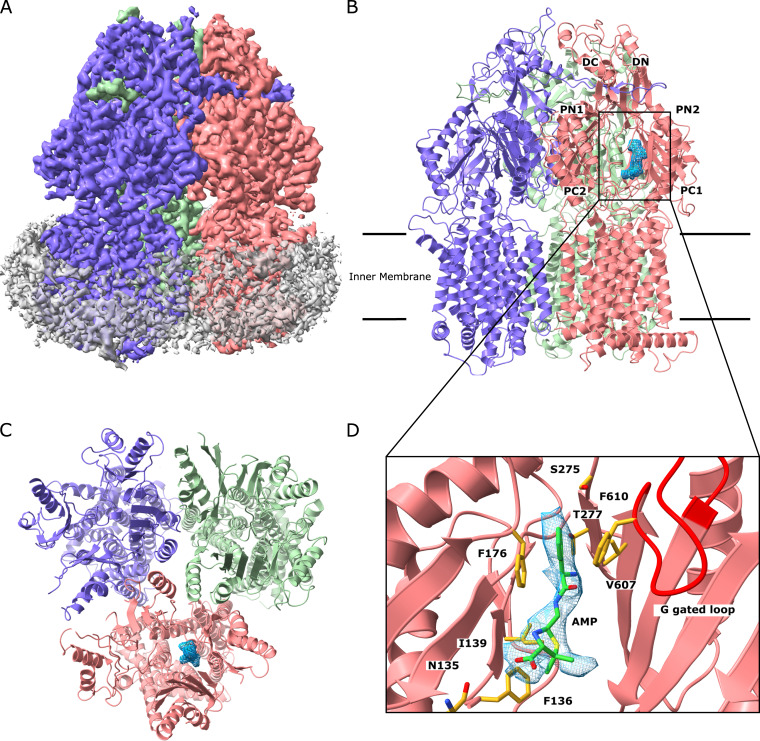
Cryo-EM structure of the MtrD_CR103_ multidrug efflux pump bound with hydrolyzed, decarboxylated ampicillin (Amp). (A) Side view of the sharpened cryo-EM map of the MtrD_CR103_ efflux pump in a lipid nanodisc. The three MtrD_CR103_ protomers are colored slate (“access” protomer), dark pink (“binding” protomer), and light green (“extrusion” protomer). Density contributed by the nanodisc is in pale gray. (B) Ribbon diagram of the MtrD_CR103_ trimer viewed from the membrane plane with the distal drug binding site displaying the density of bound Amp (blue). The access, binding, and extrusion protomers are colored slate, dark pink, and light green, respectively. (C) Ribbon diagram of the MtrD_CR103_ trimer viewed from the top of the periplasmic domain with the density of bound Amp (blue). The access, binding, and extrusion protomers are colored slate, dark pink, and light green, respectively. (D) Enlarged view of the Amp binding site. Residues that participate in Amp binding are in yellow sticks.

In order to elucidate the structure of MtrD bearing mosaic-like sequences and to understand how these pumps elevate the level of resistance to antibiotics, we used the CR.103 *mtrD* gene sequence to produce recombinant MtrD_CR103_ in Escherichia coli. We expressed recombinant MtrD_CR103_ by cloning the *mtrD_CR103_* sequence into the E. coli expression vector pET15b, with a 6×His tag at the C terminus to generate pET15bΩ*mtrD_CR103_*. This MtrD_CR103_ protein was overproduced in E. coli BL21(DE3)Δ*acrB* cells and purified using an Ni^2+^-affinity column. We reconstituted the purified MtrD_CR103_ pump into lipidic nanodiscs and determined its structure using single-particle cryo-electron microscopy (cryo-EM) (see Fig. S1 in the supplemental material). The reconstituted sample led to a cryo-EM map at a nominal resolution of 3.02 Å (Fig. S1, Table 2 and Fig. 1), allowing us to obtain a structural model of this pump. Additional densities, corresponding to the belt formed by nanodiscs, were found to encircle the transmembrane region of trimeric MtrD_CR103_. The full-length MtrD_CR103_ protein consists of 1,067 amino acids. Residues 1 to 1042 are included in our final model.

**TABLE 2:** Cryo-EM data collection, processing, and refinement statistics^*a*^

Parameter	Value	
	Ampicillin	Erythromycin
Data collection and processing		
Magnification (×)	105,000	81,000
Voltage (kV)	300	300
Electron microscope type	Krios-GIF-K2	Krios-GIF-K3
Defocus range (μm)	−1.0 to −2.5	−1.0 to −2.5
Total exposure time (s)	9	3.3
Energy filter width (eV)	20	20
Pixel size (Å)	1.1	1.08
Total dose (e^−^/Å^2^)	40	50
No. of frames	40	40
Does rate (e^−^/Å^2^/physical pixel)	5.4	17.7
No. of initial micrographs	2,033	8,528
No. of initial particle images	688,544	7,769,806
No. of final particle images	81,108	1,507,208
Symmetry	C1	C1
Resolution (Å)	3.02	2.72
FSC threshold	0.143	0.143
Map resolution range (Å)	2.85 to 9.98	2.38 to 7.06
Refinement		
Model resolution cutoff (Å)	3.02	2.72
Model composition		
No. of protein residues	3,131	3,122
No. of ligands	18	24
RMSD^*b*^		
Bond lengths (Å)	0.005	0.005
Bond angles (°)	0.790	0.644
Validation		
MolProbity score	1.65	1.91
Clash score	8.34	19.74
Poor rotamers (%)	0	0
Ramachandran plot (%)		
Favored	96.77	97.40
Allowed	3.23	2.60
Disallowed	0	0
CC^*c*^ mask	0.80	0.83
CC box	0.74	0.73
CC vol	0.78	0.83

a The dataset is MtrD reconstituted in nanodiscs (1E3D1).

b Root mean square deviation.

c Correlation coefficient.

10.1128/mBio.00996-20.1 FIG S1 Cryo-EM structure of the MtrD_CR103_ efflux pump in complex with hydrolyzed, decarboxylated ampicillin. (A) Representative 2D classes. (B) Data processing flowchart with particle distributions. A red box indicates the class used for further refinement. (C) Fourier shell correlation (FSC) curves showing resolution of 3.02 Å. (D) Density map of the hydrolyzed, decarboxylated ampicillin-bound MtrD trimer. The “access”, “binding,” and “extrusion” protomers are colored slate, dark pink, and light green, respectively. Density contributed by the nanodisc is colored gray. Download FIG S1, PDF file, 1.7 MB. Copyright © 2020 Lyu et al. 2020 Lyu et al. This content is distributed under the terms of the Creative Commons Attribution 4.0 International license.

The cryo-EM structure of MtrD_CR103_ revealed that this multidrug efflux pump adopts the overall fold of hydrophobe-amphiphile efflux (HAE)-RND-type proteins and forms a homotrimer (15, 21, 23–25, 28). Each protomer contains 12 transmembrane helices (TM1 to TM12) and a large periplasmic domain, which can be divided into six subdomains (PN1, PN2, PC1, PC2, DN, and DC) (Fig. 1). As expected, subdomains PC1 and PC2 create a periplasmic cleft, which would allow substrates to enter the pump via the periplasm. Deep inside the cleft, it contains proximal and distal multidrug recognition sites separated by the gate G-loop. Substrates that enter the periplasmic cleft would likely be sequentially bound at the proximal and then distal sites before extrusion.

**FIG 2:**
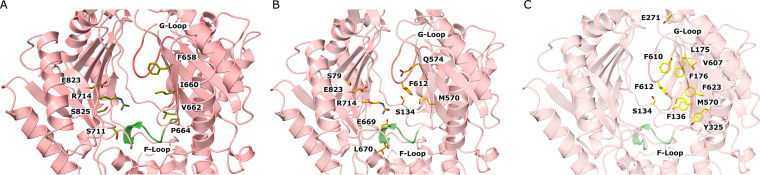
The periplasmic multidrug binding sites of MtrD_CR103_. (A) The periplasmic cleft entrance. Residues that may be important for selectivity are shown as dark yellow sticks. (B) The proximal drug binding site. The MtrD_CR103_ residues that are conserved with those for AcrB are in orange sticks. (C) The distal drug binding site. The MtrD_CR103_ residues that are conserved with those for AcrB are in light yellow sticks. The F loop and G loop are colored green and red, respectively, in A, B, and C.

The entrance of the MtrD_CR103_ periplasmic cleft is surrounded with residues F658, I660, V662, P664, S711, R714, E823, and S825 (Fig. 2A). Although several of these residues are not conserved among the HAE-RND efflux pumps, they may play a role in substrate specificity and selectivity. Indeed, within the periplasmic cleft entrance of P. aeruginosa MexB and E. coli AcrB, R716 of MexB (29) and R717 of AcrB (30) have been shown to be important for substrate specificity, suggesting that the corresponding arginine R714 of MtrD_CR103_ may also be a critical residue.

Like AdeB and AcrB, a flexible F-loop (^665^PPILELGN^672^) is found to connect the periplasmic cleft entrance and proximal multidrug binding site of MtrD_CR103_. In AcrB, it has been shown that a conserved isoleucine (I671) of the F-loop is important for drug selectivity (31). Thus, it is expected that the corresponding conserved I667 residue of MtrD_CR103_ is necessary for the pump’s function.

It has been reported that there are at least 22 amino acids making up the proximal binding site of the AcrB multidrug efflux pump (32). Eleven of these residues are conserved between AcrB and MtrD_CR103_. These MtrD_CR103_ residues are S79, S134, M570, Q574, F612, E669, L670, G671, R714, G717, and E823 (Fig. 2B), which may play an important role for drug recognition.

The composition of the MtrD_CR103_ gate G-loop is ^609^GFSFSGS^615^, where the glycines are understood to be critical and provide flexibility for the G-loop to swing the bound drug from proximal to distal binding sites (22, 32). Molecular dynamics simulations in AcrB also suggested that the phenylalanine residues of the G-loop may be important for the process of transfer and stabilizing substrate binding (32).

The distal multidrug binding site is quite extensive. It has been shown that at least 23 amino acids are involved in forming the distal site of AcrB (21, 32). Of these 23 residues, 11 are conserved between MtrD_CR103_ and AcrB. These 11 MtrD_CR103_ residues are S134, F136, L175, F176, E271, Y325, M570, V607, F610, F612, and F623 (Fig. 2C). Interestingly, mutations on these five phenylalanine residues, including F136, F176, F610, F612, and F623, have been shown to reduce resistance to different antimicrobials (33). In addition, a hydrophobic patch with a strong impact on drug binding is found in the AcrB distal site (32). In MtrD_CR103_, the composition of the distal hydrophobic patch is F176, V607, and F610. These residues are potentially critical for contacting the bound drugs.

Interestingly, the cryo-EM structure of MtrD_CR103_ indicates that this multidrug efflux pump forms an asymmetric trimer of which the three protomers are distinct and display different conformational states (Fig. 1A, B, and C). This structure is very different from the cryo-EM structure of the Acinetobacter baumannii AdeB multidrug efflux pump (23), where the three apo-protomers have an identical conformation and form a symmetrical trimer. Each protomer of AdeB prefers a transient state in which the periplasmic cleft created by subdomains PC1 and PC2 is closed in conformation. Previously, we determined a crystal structure of MtrD from N. gonorrhoeae strain PID332, designated MtrD_PID332_, and found that the three MtrD_PID332_ protomers are identical in structure in the homotrimer (15). Each MtrD_PID332_ protein within the symmetrical trimer displays a transient conformational state where the periplasmic cleft remains open.

Like the structure of asymmetric AcrB (21, 34), the conformations of the three MtrD_CR103_ protomers can be classified as the “access,” “binding,” and “extrusion” forms. Unexpectedly, an extra density was found within the distal drug-binding site of the binding protomer of MtrD_CR103_, indicating that our structure of MtrD_CR103_ is bound by a fortuitous ligand. However, there were no noticeable extra densities within the drug-binding sites of the access and extrusion protomers. The shape of this extra density is compatible with a hydrolyzed, decarboxylated ampicillin (Amp) antibiotic with its four-member β-lactam ring open (Fig. 1B and C). This is not surprising, as we supplemented with 100 μg/ml ampicillin in Luria-Bertani (LB) broth to grow E. coli BL21(DE3)Δ*acrB*/pET15bΩ*mtrD_CR103_* cells for overproducing the MtrD_CR103_ protein. The nature of the protein-substrate interaction is mostly hydrophobic. Within 4.5 Å of this bound deactivated Amp, there are five hydrophobic residues, including F136, I139, F176, V607, and F610, which provide hydrophobic interaction at this distal site to stabilize substrate binding. In addition, N135, S275, and T277 are involved and perform an electrostatic interaction to anchor this inactive drug (Fig. 1D). Interestingly, a positively charged residue R174 is found within the vicinity of this Amp binding site. It is expected that the guanidino group of this R174 residue may participate in making additional contact with other antibiotics, including the active form of β-lactams.

### Structure of the MtrD_CR103_-erythromycin complex.

The crystal structure of AcrB bound with the Ery indicated that this macrolide was anchored within the proximal drug binding site of this multidrug efflux pump, where one phenylalanine, one leucine, three serines, one threonine, one lysine, and one aspartate at the proximal site are responsible for the binding (22). A subsequent study was performed by removing the G-loop, which divides the proximal and distal multidrug binding sites of the pump (35). Again, X-ray diffraction data indicated that Ery was bound within the proximal pocket, and no evidence of Ery bound to the distal site was found for wild-type AcrB or the G-loop variant (35). It appears that Ery may prefer to occupy the proximal pocket instead of the deeper drug binding site at the distal pocket, possibly because of the large size of this drug.

**FIG 3:**
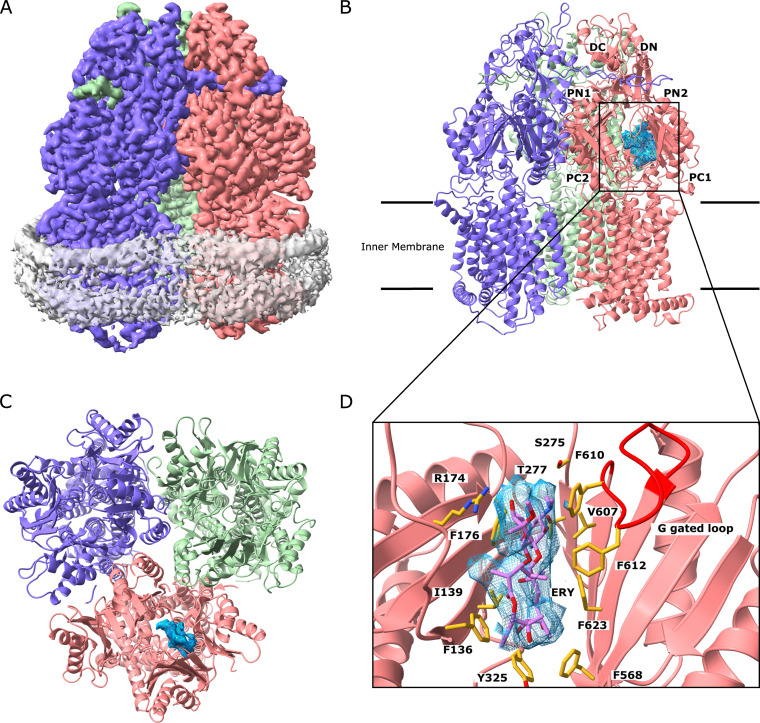
Cryo-EM structure of the MtrD_CR103_ multidrug efflux pump bound with erythromycin (Ery). (A) Side view of the sharpened cryo-EM map of the MtrD_CR103_ efflux pump in a lipid nanodisc. The three MtrD_CR103_ protomers are colored slate (“access” protomer), dark pink (“binding” protomer), and light green (“extrusion” protomer). Density contributed by the nanodisc is in pale gray. (B) Ribbon diagram of the MtrD_CR103_ trimer viewed from the membrane plane with the distal drug binding site displaying the density of bound Amp (blue). The access, binding, and extrusion protomers are colored slate, dark pink, and light green, respectively. (C) Ribbon diagram of the MtrD_CR103_ trimer viewed from the top of the periplasmic domain with the density of bound Ery (blue). The access, binding, and extrusion protomers are colored slate, dark pink, and light green, respectively. (D) Enlarged view of the Ery binding site. Residues that participate in Ery binding are in yellow sticks.

To elucidate how MtrD_CR103_ recognizes macrolide antibiotics in solution, we incubated a 2-μM MtrD_CR103_-nanodisc sample with 10 μM Ery for 2 hours to form the MtrD_CR103_-Ery complex. We determined a cryo-EM structure of this complex to a resolution of 2.72 Å (Fig. 3; see Fig. S2 in the supplemental material; Table 2). The overall structure of MtrD_CR103_-Ery is almost identical to that of Amp bound (labeled as MtrD_CR103_-Amp), with the three protomers displaying the access, binding, and extrusion forms (Fig. 3A, B, and C). Superimposition of the MtrD_CR103_-Ery and MtrD_CR103_-Amp trimers results in an overall root mean square deviation (RMSD) of 0.472 Å. In the binding protomer of MtrD_CR103_, the cryo-EM images depict that an additional large density corresponding to the bound Ery drug was found at the distal multidrug binding site. It is somewhat surprisingly that the observed large Ery molecule is anchored within this deep pocket. It is possible that the protein molecules are more flexible to accommodate the bound drug in solution instead of within the crystal lattice. The binding of Ery is extensive; within 4.5 Å of this bound drug, there are seven aromatic, two hydrophobic, two polar, and one positively charged residues that provide aromatic stacking and hydrophobic and electrostatic interactions to facilitate Ery binding. These important binding residues are F136, I139, R174, F176, S275, T277, Y325, F568, V607, F610, F612, and F623 (Fig. 3D). Many of these interactions are shared between MtrD_CR103_-Amp and MtrD_CR103_-Ery; however, the larger Ery molecule interacts with four additional residues. The MtrD_CR103_ periplasmic binding cleft is able to accommodate Ery and forms additional electrostatic and hydrophobic contacts with R174, Y325, F568, and F623. The ability of the periplasmic binding cleft to accommodate both Amp and Ery suggests that the MtrD_CR103_ efflux pump is effective at binding and extruding a broad spectrum of antimicrobial compounds.

10.1128/mBio.00996-20.2 FIG S2 Cryo-EM structure of the MtrD_CR103_ efflux pump in complex with erythromycin. (A) Representative 2D classes. (B) Data processing flowchart with particle distributions. A blue box indicates the class used for further refinement. (C) Fourier shell correlation (FSC) curves showing resolution of 2.72 Å. (D) Density map of the erythromycin-bound MtrD trimer. The “access”, “binding,” and “extrusion” protomers are colored slate, dark pink, and light green, respectively. Density contributed by the nanodisc is colored gray. Download FIG S2, PDF file, 2.0 MB. Copyright © 2020 Lyu et al. 2020 Lyu et al. This content is distributed under the terms of the Creative Commons Attribution 4.0 International license.

### The proton relay network.

**FIG 4:**
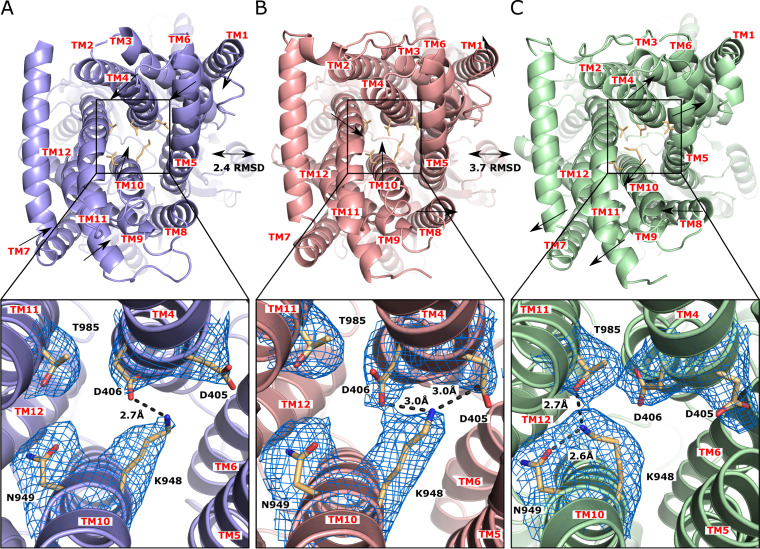
The proton-relay network of the MtrD_CR103_ multidrug efflux pump. (A) The “access” state of MtrD_CR103_. In this conformational state, the “proton sweeper” K948 forms a hydrogen bond with D406. The densities of residue side chains (D405, D406, K948, N949, and T985), which form the proton-relay network, are in blue meshes. (B) The “binding” state of MtrD_CR103_. In this conformation, K948 forms hydrogen bonds with both D405 and D406. (C) The “extrusion” state of MtrD_CR103_. In this conformation, K948 forms hydrogen bonds with N949 and T985. Superimposition of the structures of the access and binding states results in an RMSD of 2.4 Å, whereas the superimposition between the binding and extrusion states gives rise to an RMSD of 3.7 Å.

The high-quality density of our cryo-EM map unambiguously depicts the side chain positions of these conserved amino acids, allowing us to elucidate the transfer of protons within this proton-relay network. It is known that the proton motive force (PMF) powers RND efflux pumps to extrude drugs from the periplasmic domain. In the transmembrane domain of MtrD_CR103_, residues D405, D406, K948, N949, and T985 are conserved and form the proton relay network (Fig. 4). These residues are likely responsible for proton translocation from the periplasm to the cytoplasm and generate the PMF necessary to extrude drugs from the cell. The influx of protons to the cytosol and efflux of drugs out of the cell should be synchronized and coupled with each other. In the “access state,” the conserved residues K948 and D406 closely interact with each other. The side chain nitrogen of K948 forms a single hydrogen bond of 2.7 Å to the carboxylate oxygen of the side chain of D406 (Fig. 4A). Upon binding a drug in the periplasmic domain, a major allosteric trigger is switched, causing a shift in the protomer to the binding form. Interestingly, the conformation of the proton relay network is also quite distinct at this state. We observed that the side chain of the conserved residue D405 moves closer to K948, allowing this conserved lysine to form an additional hydrogen bond with D405. In this binding state, the hydrogen-bonded distances between the side chain nitrogen atom of K948 and carboxylate oxygens of D405 and D406 are both 3.0 Å (Fig. 4B). It is likely that the side chain carboxylate oxygen of D405 donates its proton to form the hydrogen bond with the side chain nitrogen of K948 at this state. To advance the transport cycle, the MtrD_CR103_ membrane protein probably needs to switch its conformation to the extrusion form, where the periplasmic cleft is closed. At this transient state, the side chain of K948 points toward the conserved polar residues N949 and T985, enabling this positively charged residue to form 2.6-Å-long and 2.7-Å-long hydrogen bonds with N949 and T985, respectively (Fig. 4C). In this conformation, we believe that K948 offers the proton released from D405 to form one of these two hydrogen bonds. The dynamic change of this hydrogen bonding network may promote the transfer of protons from the periplasm to cytoplasm that creates the PMF to energize the drug efflux process. It is likely that K948 is responsible for transferring protons from D405 to N949 or T985 to advance this transfer process. It appears that K948 plays a major role in sweeping protons from the periplasm to the cytoplasm. Because of the importance of K948 in proton transfer, we designate this residue as a “proton sweeper.” During the process of proton translocation, D406 may participate in stabilizing different transient states by interacting with the critical K948 residue.

### The lipid binding sites.

The cryo-EM structure of the MtrD_CR103_-Amp complex indicates that at least 17 phosphatidylethanolamine (PE) lipids are anchored within the transmembrane domain. Likewise, the cryo-EM structure of the MtrD_CR103_-Ery complex depicts that there are 23 PE lipids that are similarly oriented as the lipids in the Amp-bound structure. The modes of binding and orientations of the bound PE lipids are very similar for both structures, although the MtrD_CR103_-Ery complex is observed to have six more bound lipid molecules.

**FIG 5:**
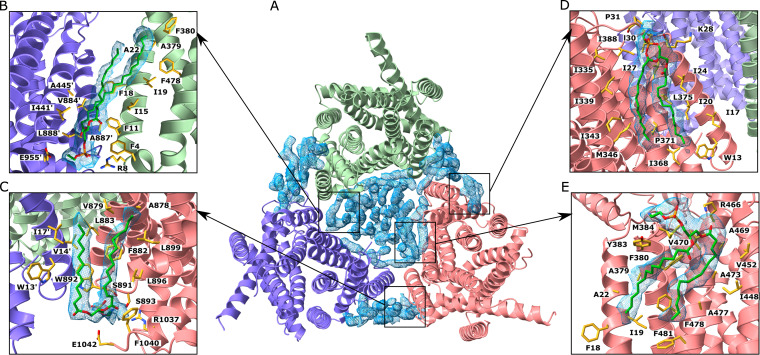
Densities of bound lipids at the transmembrane domain of the MtrD_CR103_ multidrug efflux pump. (A) Viewed from the periplasmic side of the MtrD_CR103_-Ery trimer. There are 23 PE lipid molecules attached to the transmembrane region of this membrane protein. Densities of the bound PE lipids are in blue meshes. (B–D) The three distinct lipid binding sites at the interface between MtrD_CR103_ protomers. The bound PE lipids are in green sticks. Residues involved in lipid binding are in yellow sticks. (E) The lipid binding site at the interior wall of the central cavity of a MtrD_CR103_ protomer. The bound PE lipid is in green sticks. Residues involved in lipid binding are in yellow sticks.

The PE lipids are found to anchor within the large central cavity of the MtrD_CR103_ trimer in the transmembrane region. In addition, some lipid molecules are also identified at protomer-protomer interfaces between subunits of the protein (Fig. 5A). It is observed that several lipids specifically interact with the MtrD_CR103_ protomers. For example, at least three lipid molecules are bound at distinct binding sites formed at the interface between MtrD_CR103_ protomers, where the lipids are found to directly contact the protein molecules (Fig. 5B and C and D). Additionally, each MtrD_CR103_ molecule by itself forms a PE binding site, which specifically anchors a lipid molecule (Fig. 5E), and this PE-binding site is conserved with that of the AdeB multidrug efflux pump (23).

### Drug susceptibility of mutant MtrD-bearing isogenic strains.

Within the periplasmic entrance and proximal and distal sites of MtrD_CR103_, we noticed that there are several charged amino acids, including R174, E669, R714, and E823, which may be critical for the specificity of the pump. We used site-directed mutagenesis to cause single-amino acid changes at residues 174, 669, and 714. These mutated *mtrD* genes, as well as S821A, K823E, and S825A mutant or WT genes, were then transformed into strain KH14 so as to replace the *mtrD::kan* sequence; as described above, KH14 is hypersusceptible to antimicrobials exported by the MtrCDE efflux pump (14). As is shown in Table 1, compared with KH14 bearing the introduced WT *mtrD*, site-directed mutations at positions 174, 669, 714, and 823 influenced levels of antimicrobial resistance. Compared to the WT MtrD, a mutation in the distal binding domain (R174Q) was found to selectively increase the MIC of ethidium bromide (EtBr), which was in contrast to the impact of the K823E entrance site mutation that seemed selective for macrolides. In contrast to these mutations, the proximal binding site-located E669G mutation decreased (2-fold) gonococcal resistance to both macrolides and polymyxin B compared with the WT protein. While we cannot presently distinguish whether the E669G mutation directly influences efflux activity or pump stability, the WT levels of resistance to CV and TX-100 (Table 1) favors the former possibility. With respect to the R174G and S825A mutations, only the R714G change influenced antimicrobial resistance, and this was also selective for macrolides. As with strain KH15 K823E, introduction of the R714G mutation could also further increase macrolide resistance compared with the parental strain KH15. Interestingly, this mutation, as well as K823E, enhanced gonococcal resistance to polymyxin B, a model cationic antimicrobial peptide, when present in *mtrCDE* overexpressed strain KH15. For verification of the results obtained using the agar dilution method, we also assessed changes in gonococcal susceptibility to two antimicrobials (Azi and EtBr) due to site-directed MtrD mutations using a population antimicrobial assay; this assay employed spotting 5-μl samples of 1:5 dilutions of isogenic strains onto Gonococcal Medium Base (GCB) agar containing indicated concentrations of the test antimicrobial. In agreement, the E669G mutation resulted in increased susceptibility to both Azi and EtBr (see Fig. S3 in the supplemental material). In contrast, when present in strain KH15, the R714G and K823E mutations increased gonococcal resistance to both Azi and EtBr (see Fig. S4 in the supplemental material). Importantly, we predict that gonococcal strains bearing both the *mtrR* promoter and amino acid changes at MtrD positions 714 or 823 could lead to clinically significant levels of Azi nonsusceptibility resistance (27).

10.1128/mBio.00996-20.3 FIG S3 Population analysis. Shown is the growth of 5 microliters of spotted dilutions (undiluted to 1:3,125) of gonococcal strains containing WT or site-directed *mtrD* mutations that were transformed into strain KH14 (FA19 *mtrD::kan*) on GCB agar plates containing no antimicrobial or Azi (0.03 and 0.125 μg/ml) or EtBr (0.25, 1.0, or 2.0 μg/ml). All plates were incubated at 37°C under 5% (v/v) carbon dioxide conditions for 48 h before being photographed. Download FIG S3, PDF file, 0.1 MB. Copyright © 2020 Lyu et al. 2020 Lyu et al. This content is distributed under the terms of the Creative Commons Attribution 4.0 International license.

10.1128/mBio.00996-20.4 FIG S4 Population analysis. Shown is the growth of 5 microliters of spotted dilutions (undiluted to 1:3,125) of gonococcal strains containing site-directed *mtrD* mutations that were transformed into strain KH15 (FA19 with the single bp deletion in the *mtrR* promoter) on GCB agar plates containing no antimicrobial or Azi (0.5 and 2.0 μg/ml) or EtBr (4.0 or 8.0 μg/ml). All plates were incubated at 37°C under 5% (v/v) carbon dioxide conditions for 48 h before being photographed. Download FIG S4, PDF file, 0.1 MB. Copyright © 2020 Lyu et al. 2020 Lyu et al. This content is distributed under the terms of the Creative Commons Attribution 4.0 International license.

## DISCUSSION

The emergence and spread of drug-resistant N. gonorrhoeae have significantly challenged our efforts to reliably and effectively treat gonorrhea. There is an increasing trend of resistance to Azi and Cro, which constitute the last favored course of dual empirical therapy, making the problem even more alarming. One major mechanism that N. gonorrhoeae utilizes to mediate antimicrobial resistance is the involvement of multidrug efflux pumps, which effectively lower intracellular drug concentrations well below toxic levels. We and others recently discovered that certain clinical isolates of N. gonorrhoeae are able to acquire mosaic-like *mtrD* efflux pump genes to enhance the MtrD pump activity, resulting in elevating the level of resistance to the Azi and Ery macrolides (17, 20).

Our cryo-EM data reveal the Amp and Ery binding sites of MtrD_CR103_. These two drug binding sites overlap each other. It appears that MtrD_CR103_ tends to use the conserved phenylalanines F136, F176, F610, F612, and F623 to bind these drugs. These phenylalanines have been found to be important for drug resistance and involved in substrate transport (33). Hence, aromatic and hydrophobic interactions may be general mechanisms for MtrD_CR103_ to recognize multiple antimicrobials.

One major target for macrolides is the large 50S ribosomal subunit of bacterial 70S ribosomes. These antibiotics inhibit the translocation of the ribosome along the mRNA chain and halt bacterial protein synthesis. The HAE-type multidrug efflux pumps extrude these antibiotics, disallowing them to damage bacterial cells. It was not initially clear if efflux pumps and ribosomes bind macrolides in a similar fashion. However, our cryo-EM structural data indicate that MtrD_CR103_ uses six phenylalanine and one tyrosine amino acids, which provide aromatic and hydrophobic interactions to anchor Ery at the distal binding site. Indeed, there is considerable similarity between this drug binding mode and the one found in the Staphylococcus aureus 70S ribosome (36), where the large 50S subunit utilizes the aromatic rings of eight nucleotides to contact the Ery macrolide.

MtrD_CR103_ is a PMF-dependent pump that functions via an antiport mechanism. Coupled with the export of drug molecules toward the periplasm, protons have to flow into the cytoplasm to energize this efflux process. Our cryo-EM maps unambiguously depict the dynamic changes of residue side chains, including D405, D406, K948, N949, and T985, which are involved in the proton-relay network within the transmembrane helices. The proton sweeper K948 should be deemed necessary to transfer protons across the cytoplasmic membrane for energy coupling.

Based on the structural information, there are several charged residues, such as R174, E669, R714, and E823, participating in forming substrate binding sites within the periplasmic domain. Among these residues, E823 was found in an N. gonorrhoeae clinical isolate (CDC2) possessing mosaic-like *mtr* sequences (17). This residue is a lysine in the WT protein expressed by the antibiotic-sensitive strain FA19. This mutation likely results in enhancing the recognition of antimicrobials, leading to increased efflux activity and reduced susceptibility to antimicrobials. Our cryo-EM structures depict that both R714 and E823 are engaged in the entrance and proximal sites, which are probably responsible for drug recognition and discrimination. These two charged residues appear to provide resistance to macrolides to a clinically significant level. Acquiring further structural data with a broad spectrum of antimicrobial compounds may prove useful for elucidating the binding system of this multidrug efflux pump. Combined, our studies will ultimately inform structure-guided drug design for inhibiting the critical MtrD multidrug efflux pump and other pumps to help combat the increasing threat of antibiotic resistance.

## MATERIALS AND METHODS

### Expression and purification of MtrD_CR103_.

The *mtrD_CR103_* gene, encoding the MtrD_CR103_ multidrug efflux pump, from N. gonorrhoeae CR.103 was cloned into the pET15b expression vector in frame with a 6×His tag at the C terminus. The resulting pET15bΩ*mtrD_CR103_* plasmid was confirmed by the Sanger method of DNA sequencing. The MtrD_CR103_ protein was overproduced in Escherichia coli BL21(DE3)Δ*acrB* cells, which harbor a deletion in the chromosomal *acrB* gene. Cells were grown in 6 liters of LB medium with 100 μg/ml ampicillin at 37°C. When the optical density at 600 nm (OD_600_) reached 0.5, the culture was treated with 0.2 mM isopropyl-β-d-thiogalactopyranoside (IPTG) to induce *mtrD_CR103_* expression. Cells were then harvested within 3 h of induction. The collected bacteria were resuspended in low salt buffer (100 mM sodium phosphate [pH 7.4], 10% glycerol, 5 mM ethylenediaminetetraacetic acid [EDTA], and 1 mM phenylmethanesulfonyl fluoride [PMSF]) and then disrupted with a French pressure cell. The membrane fraction was collected and washed twice with high salt buffer (20 mM sodium phosphate [pH 7.4], 2 M KCl, 10% glycerol, 5 mM EDTA, and 1 mM PMSF) and once with 20 mM HEPES-NaOH buffer (pH 7.5) containing 1 mM PMSF. The membrane protein was then solubilized in 2% (wt/vol) n-dodecyl-β-d-maltoside (DDM). Insoluble material was removed by ultracentrifugation at 100,000 × *g*. The extracted protein was then purified with an Ni^2+^-affinity column. The purity of the MtrD_CR103_ protein (>95%) was judged using SDS-PAGE stained with Coomassie brilliant blue. The purified protein was dialyzed against 20 mM Na-HEPES (pH 7.5) and concentrated to 8.2 mg/ml (72 μM) in a buffer containing 20 mM Na-HEPES (pH 7.5) and 0.05% DDM.

### Nanodisc preparation.

To assemble MtrD_CR103_ into nanodiscs, a mixture containing 20 μM MtrD_CR103_, 45 μM MSP (1E3D1), and 930 μM E. coli total extract lipid was incubated for 15 minutes at room temperature. Afterward, 0.8-mg/ml prewashed Bio-Beads (Bio-Rad) was added. The resultant mixture was incubated for 1 h on ice, followed by overnight incubation at 4°C. The protein-nanodisc solution was filtered through 0.22-μm nitrocellulose-filter tubes to remove the Bio-Beads. To separate free nanodiscs from MtrD_CR103_-loaded nanodiscs, the filtered protein-nanodisc solution was purified using a Superose 6 column (GE Healthcare) equilibrated with 20 mM Tris-HCl (pH 7.5) and 100 mM NaCl. Fractions corresponding to the size of the trimeric MtrD_CR103_-nanodisc complex were collected for cryo-EM.

### Electron microscopy sample preparation.

The trimeric MtrD_CR103_ nanodisc sample was concentrated to 0.7 mg/ml (2 μM) and applied to glow-discharged holey carbon grids (Quantifoil Cu R1.2/1.3, 300 mesh), blotted for 15 s, and then plunge-frozen in liquid ethane using a Vitrobot (Thermo Fisher). The grids were transferred into cartridges. The images were recorded at 1- to 2.5-μm defocus on a K2 summit direct electron detector (Gatan) with superresolution mode at nominal 105 K magnification, corresponding to a sampling interval of 1.10 Å/pixel (superresolution, 0.55 Å/pixel). Each micrograph was exposed for 9 s with 5.40 e^−^/s/physical pixel dose rate (total specimen dose, 40 e^−^/A^2^), and 40 frames were captured per specimen area using Latitude.

For the MtrD_CR103_-Ery sample, a 2-μM MtrD_CR103_-nanodisc sample was incubated with 10 μM Ery for 2 hours to form the MtrD_CR103_-Ery complex. The procedures for making cryo-EM grids were the same as above. The images were recorded at −1- to −2.5-μm defocus on a K3 summit direct electron detector (Gatan) with counting mode at nominal ×81,000 magnification, corresponding to a sampling interval of 1.08 Å/pixel (superresolution, 0.54 Å/pixel). Each micrograph was exposed for 3.3 s with 17.7 e^−^/s/physical pixel dose rate (total specimen dose, 50 e^−^/Å^2^), and 40 frames were captured per specimen area using Latitude.

### Data collection and processing.

The image stacks in the superresolution model were aligned using cryoSPARC (37). The contrast transfer function (CTF) parameters of the micrographs were determined using Gctf (38). After manual inspection and sorting to discard poor images, ∼2,000 particles were manually picked to generate templates for automatic picking. Initially, 688,544 particles were selected after autopicking in cryoSPARC (37). Several iterative rounds of 2D classifications were carried out to remove false picks and classes with unclear features, ice contamination, or carbon. The resulting 203,346 particles were used to generate a reference-free ab-initio 3D reconstruction. Two rounds of heterogeneous refinement were used, where 81,108 particles were chosen for further processing with local motion correction using cryoSPARC (37) and local CTF reestimation by Gctf (38). The nonuniform refinement followed by local focused refinement using cryoSPARC resulted in a 3.02-Å global resolution map based on the gold standard Fourier shell correlation (FSC) (Table 2; Fig. S1).

For MtrD_CR103_-Ery, 7,769,806 particles were selected after autopicking in cryoSPARC (37). After several rounds of 2D classifications, 2,102,459 particles were selected to generate three ab-initio models and then subjected to two rounds of 3D heterogeneous refinements. The resulting 1,507,208 particles were finally chosen for further processing with nonuniform and local CTF refinement (38), resulting in a 2.72-Å resolution cryo-EM structure (Table 2; Fig. S2).

### Model building and refinement.

Model building of MtrD_CR103_-Amp was based on the 3.02-Å cryo-EM map. The homology modeling structure of MtrD_CR103_ generated by the FFAS server based on the atomic coordinates of AdeB (PDB ID: 6OWS) (23) was fit into the density map using Chimera (39) The subsequent model rebuilding was performed using Coot (40). Structure refinements were performed using the phenix.real_space_refine program (41) from the PHENIX suite (42). The final atomic model was evaluated using MolProbity (43). The statistics associated with data collection, 3D reconstruction, and model refinement are included in Table 2.

The 2.72-Å resolution of the MtrD_CR103_-Ery structural model was built based on the MtrD_CR103_-Amp structure. Structural refinements were done using the same approach as above (Table 2).

### Site-directed mutagenesis of *mtrD* and antimicrobial susceptibility testing.

In order to construct single-amino acid changes in MtrD, the *mtrD* gene plus 269-bp up- and 262-bp downstream flanking regions was PCR amplified from FA19 genomic DNA using primers KH9#16 and mtrE12 with Phusion high-fidelity DNA polymerase. The PCR product was cloned into the pCR4Blunt-TOPO vector using the Blunt Topo sequencing kit (Invitrogen). The resulting vector was named pCR4Blunt-TOPO::mtrD. The Quickchange Lightning site-directed mutagenesis kit (Agilent) was used to replace specific residues (R174, E669, R714, S821, K823, and S825). Primers for site-directed mutagenesis were designed utilizing the Agilent tool (https://www.agilent.com/store/primerDesignProgram.jsp). Mutagenic constructs were transformed into E. coli XL-10 Gold cells. For allelic replacement of the chromosomal *mtrD* region, the mutagenic constructs containing the desired point mutations were transformed into strain KH14 (FA19 *mtrD*::kan). Transformants were selected on GC agar containing 0.625 μg/ml of erythromycin. DNA sequencing was used to confirm that for each strain the entire *mtrRCDE* locus contained only the desired point mutation. Antimicrobial susceptibility of strains was performed using the agar dilution method with various concentrations of selected antimicrobials (Table 1). All antimicrobial assays were performed at least in triplicate.

To further examine the effect of the single-amino acid changes on efflux, we constructed mutants in an overexpression background. KH15 is a transformant of strain FA19 which has a single-bp deletion in a 13-bp inverted repeat sequence within the *mtrR* promoter. This deletion results in overexpression of the *mtrCDE* efflux pump-encoding operon (9). The mutagenic constructs containing the desired point mutations described previously were transformed into strain FA19Str and selected on 0.25 μg/ml azithromycin. A PCR product containing the single-bp deletion was amplified from KH15 using primers CEL1 and KH9#12B. This PCR product was then transformed into the FA19Str R714G and K823E mutants. KH15 transformants were selected on 1,600 μg/ml of Triton X-100. DNA sequencing was used to confirm that for each strain the entire *mtrRCDE* locus contained only the desired point mutation and the single-bp deletion in the promoter region. Antimicrobial susceptibility of strains was performed using the agar dilution method with various concentrations of selected antimicrobials (Table 1). All antimicrobial assays were performed at least in triplicate. In other assays, a population-based assessment assay was employed that spotted 5 μl of undiluted or 1:5 dilutions of overnight GCB agar-grown cultures resuspended in GCB broth to give approximately 1 × 10^8^ CFU/ml onto GCB agar with or without Azi or EtBr. All plates were incubated at 37°C under 5% (vol/vol) CO_2_ conditions for 48 h.

### Data availability.

Atomic coordinates and structure factors of MtrD_CR103_-Amp and MtrD_CR103_-Ery have been deposited at the RCSB Protein Data Bank with accession codes 6VKS and 6VKT, respectively.
