# Supplementary material for: Cryo-EM Structures of a Gonococcal Multidrug Efflux Pump Illuminate a Mechanism of Drug Recognition and Resistance
Source: mBio. 2020 May 26;11(3):e00996-20. doi: 10.1128/mBio.00996-20 (PMC7251214; doi:10.1128/mBio.00996-20)
Supplement: FIG S2 [file mBio.00996-20-sf002.pdf]

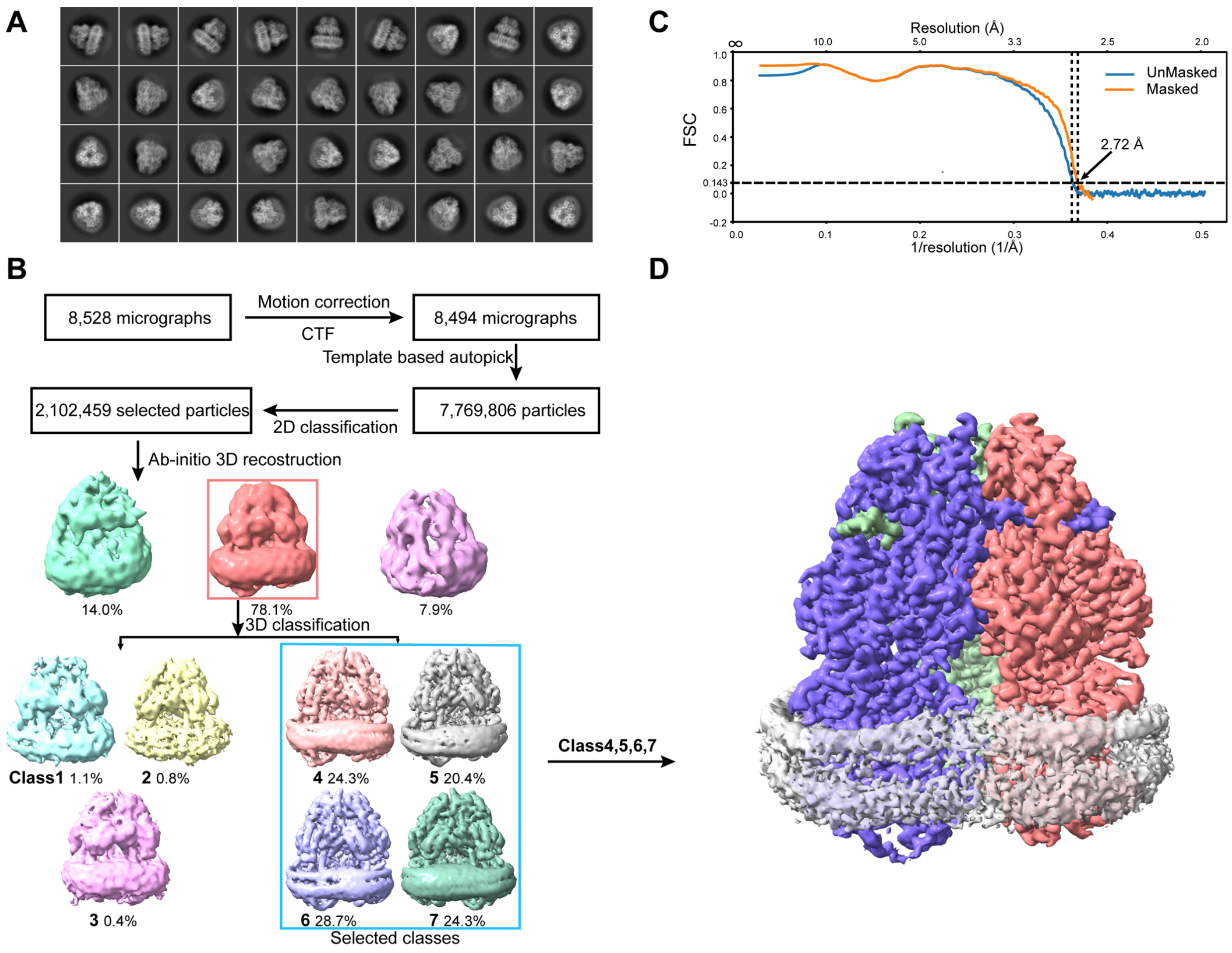

Figure S2. Cryo-EM structure of the MtrD$_{CR103}$ efflux pump in complex with erythromycin. (A) Representative 2D classes. (B) Data processing flowchart with particle distributions. A blue box indicates the class used for further refinement. (C) Fourier shell correlation (FSC) curves showing resolution of 2.72 Å. (D) Density map of the erythromycin bound MtrD trimer. The "access", "binding" and "extrusion" protomers are colored slate, dark pink and light green, respectively. Density contributed by the nanodisc is colored gray.
