# Supplementary material for: Cryo-EM Structures of a Gonococcal Multidrug Efflux Pump Illuminate a Mechanism of Drug Recognition and Resistance
Source: mBio. 2020 May 26;11(3):e00996-20. doi: 10.1128/mBio.00996-20 (PMC7251214; doi:10.1128/mBio.00996-20)
Supplement: FIG S3 [file mBio.00996-20-sf003.pdf]

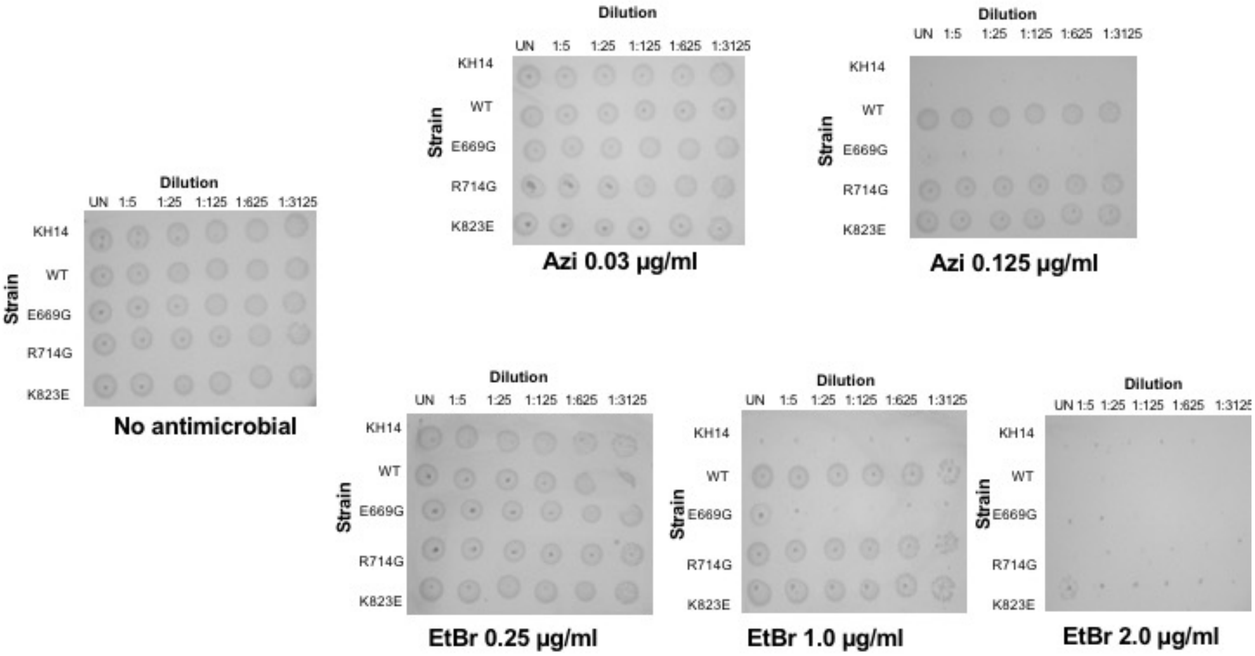

Figure S3. Population analysis. Shown are growth of 5 microliters of spotted dilutions (undiluted to 1:3,125) of gonococcal strains containing WT or site-directed *mtrD* mutations that were transformed into strain KH14 (FA19 *mtrD::kan*) on GCB agar plates containing no antimicrobial or Azi (0.03 and 0.125 μg/ml) or EtBr (0.25, 1.0 or 2.0 μg/ml). All plates were incubated at 37 ^{0}C under 5% (v/v) carbon dioxide) for 48 hr before photography.
