# Supplementary material for: Cryo-EM Structures of a Gonococcal Multidrug Efflux Pump Illuminate a Mechanism of Drug Recognition and Resistance
Source: mBio. 2020 May 26;11(3):e00996-20. doi: 10.1128/mBio.00996-20 (PMC7251214; doi:10.1128/mBio.00996-20)
Supplement: FIG S4 [file mBio.00996-20-sf004.pdf]

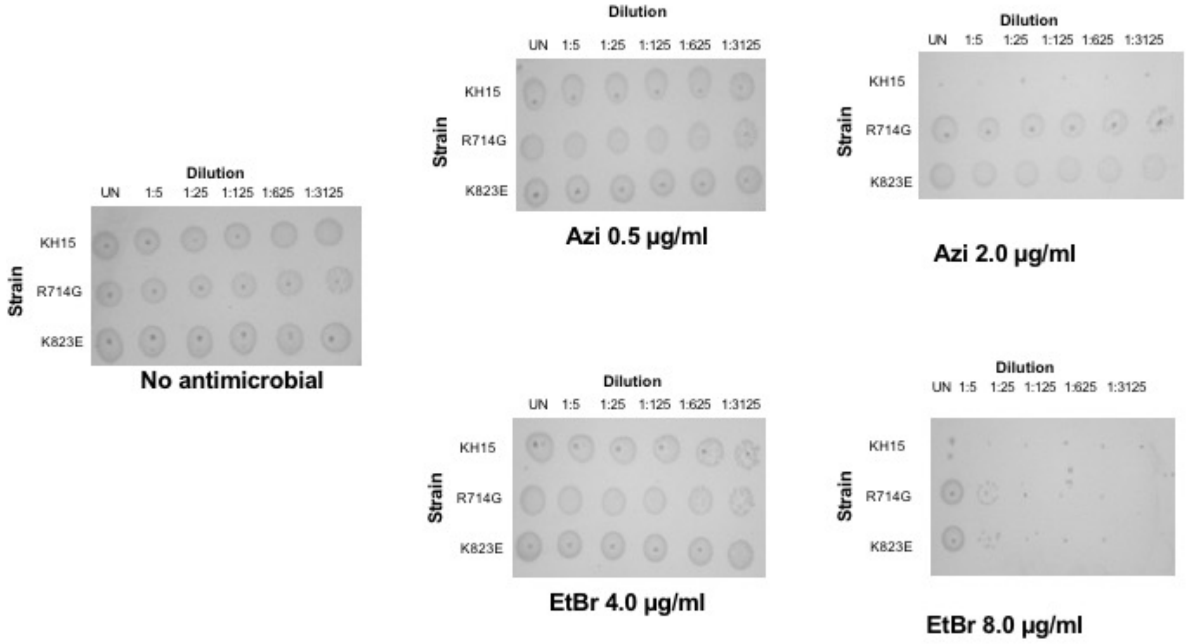

Figure S4. Population analysis. Shown are growth of 5 microliters of spotted dilutions (undiluted to 1:3,125) of gonococcal strains containing site-directed *mtrD* mutations that were transformed into strain KH15 (FA19 with the single bp deletion in the *mtrR* promoter) on GCB agar plates containing no antimicrobial or Azi (0.5 and 2.0 µg/ml) or EtBr (4.0 or 8.0 µg/ml). All plates were incubated at 37 ^{0}C under 5% (v/v) carbon dioxide) for 48 hr before photography.
